# Advances in synthetic lethality for cancer therapy: cellular mechanism and clinical translation

**DOI:** 10.1186/s13045-020-00956-5

**Published:** 2020-09-03

**Authors:** Win Topatana, Sarun Juengpanich, Shijie Li, Jiasheng Cao, Jiahao Hu, Jiyoung Lee, Kenneth Suliyanto, Diana Ma, Bin Zhang, Mingyu Chen, Xiujun Cai

**Affiliations:** 1 Department of General Surgery, Sir Run-Run Shaw Hospital, Zhejiang University, Hangzhou, 310016 China; 2 School of Medicine, Zhejiang University, Hangzhou, 310058 China; 3 Institute of Pharmaceutics, College of Pharmaceutical Sciences, Zhejiang University, Hangzhou, 310058 China; 4 Key Laboratory of Endoscopic Technique Research of Zhejiang Province, No.3 East Qingchun Road, Hangzhou, 310016 China

**Keywords:** Cancer therapy, Synthetic lethality, PARP inhibitors, DNA damage response inhibitors, DNA repair

## Abstract

Synthetic lethality is a lethal phenomenon in which the occurrence of a single genetic event is tolerable for cell survival, whereas the co-occurrence of multiple genetic events results in cell death. The main obstacle for synthetic lethality lies in the tumor biology heterogeneity and complexity, the inadequate understanding of synthetic lethal interactions, drug resistance, and the challenges regarding screening and clinical translation. Recently, DNA damage response inhibitors are being tested in various trials with promising results. This review will describe the current challenges, development, and opportunities for synthetic lethality in cancer therapy. The characterization of potential synthetic lethal interactions and novel technologies to develop a more effective targeted drug for cancer patients will be explored. Furthermore, this review will discuss the clinical development and drug resistance mechanisms of synthetic lethality in cancer therapy. The ultimate goal of this review is to guide clinicians at selecting patients that will receive the maximum benefits of DNA damage response inhibitors for cancer therapy.

## Introduction

Cancer is regarded as a complex disease with multiple genetic changes, including oncogenes, tumor suppressors, DNA repair, cancer metabolism, and genetic background, which results in excessive growth, metastasis, and drug resistance [1–3]. The advances in genome sequencing over the past decade demonstrated that tumor-specific genetic alterations and biological changes drive tumor progression, which leads to susceptibilities that could be manipulated to target tumors selectively [4]. Multiple studies have indicated that synthetic lethality is a promising approach that could improve cancer research and treatment [5, 6]. The concept of synthetic lethality originates from genetic studies in fruit flies which describes the incompatibility between pairs of alleles [7] and indicates the instance where the co-occurrence of multiple gene mutations results in cell death [8]. Unlike conventional targeted therapies, synthetic lethal therapies promote mutation indirect targeting by identifying an alternative synthetic lethal target, ranging from oncogenes to tumor suppressors, DNA repair, cancer metabolism, and even genetic background [9]. Therefore, synthetic lethal interactions have the potential to broaden the strategies of anticancer treatments and stimulate drug discovery.

Synthetic lethal therapy has been referred to as one of the most effective cancer therapies in the last decade. Poly(ADP-ribose) polymerase (PARP) inhibitors, which targeted the inhibition of particular DNA repair pathways, have become the drug based on the synthetic lethal approach that is approved for clinical use to target BRCA1/2-mutated tumors. This synthetic lethal interaction was discovered and approved as a specific and safer therapy for cancer [10, 11]. Various studies demonstrated that PARP inhibitors had promising results in clinical trials for BRCA1/2-mutant tumors, such as breast cancer, ovarian cancer, pancreatic cancer, and prostate cancer [12–16]. Moreover, with the development of new experimental and computational approaches, researchers have identified and validated several new synthetic lethal interactions. The underlying synthetic lethal interactions, mechanistic characterization, and screening approaches will raise the possibility of clinical translations and promote novel and effective synthetic lethality strategies.

This review will describe the current understandings of synthetic lethality mechanisms, advances, and challenges. The advantages and limitations of various approaches for discovering additional synthetic lethal interactions will be explored. Furthermore, recent clinical developments for DNA damage response (DDR) inhibitors and their resistance mechanisms will be discussed. Finally, we will examine the new directions and opportunities for synthetic lethality in anticancer-targeted therapy.

## Synthetic lethal mechanism

**Fig. 1:**
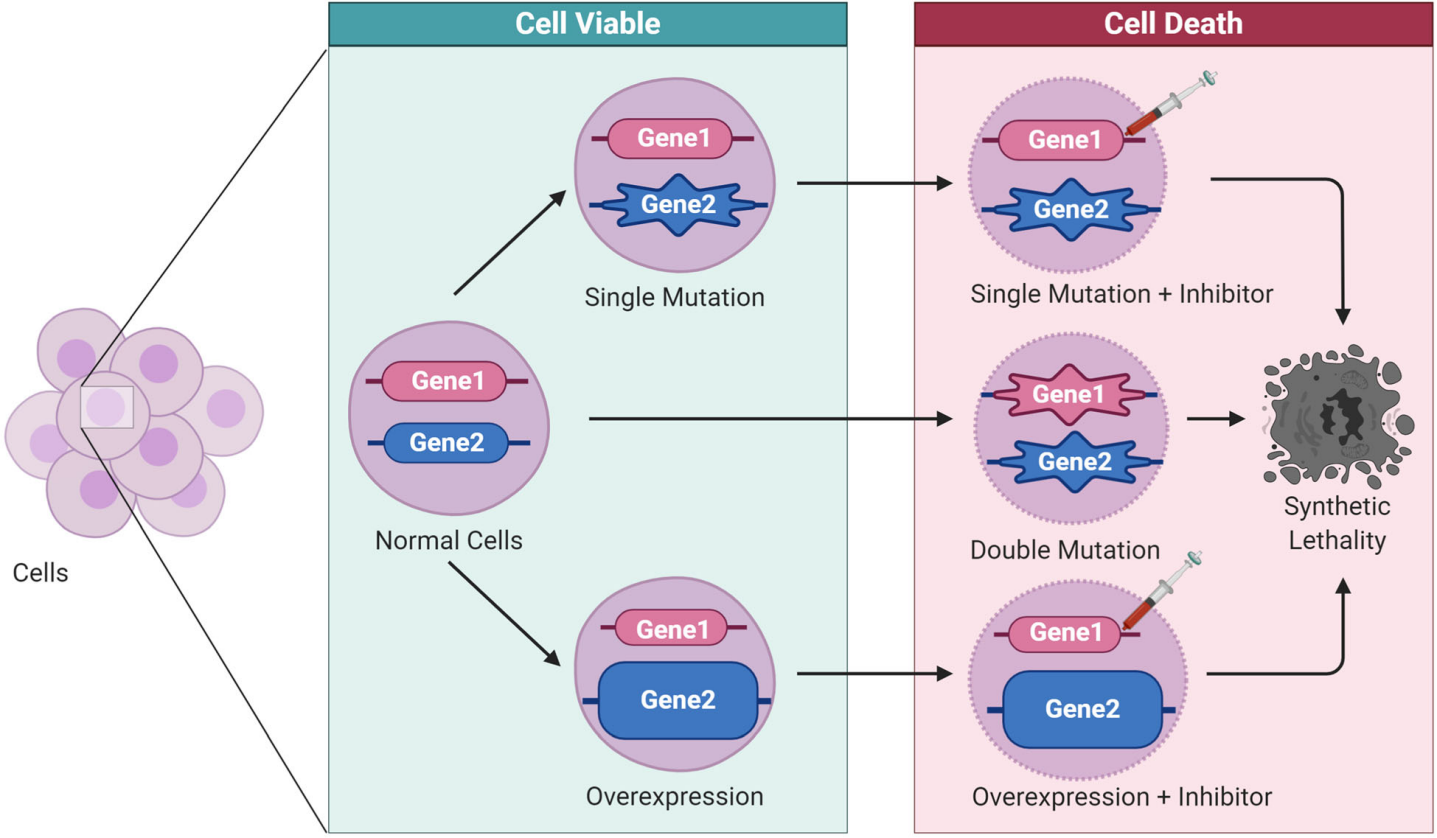
The principle of synthetic lethality. An individual genetic event is compatible with cell viability (left), whereas the co-occurrence of multiple genetic events causes cell death (right). The star represents a mutation; the large bubble represents genetic overexpression; the syringe represents DNA damage response inhibitor administration

**Fig. 2:**
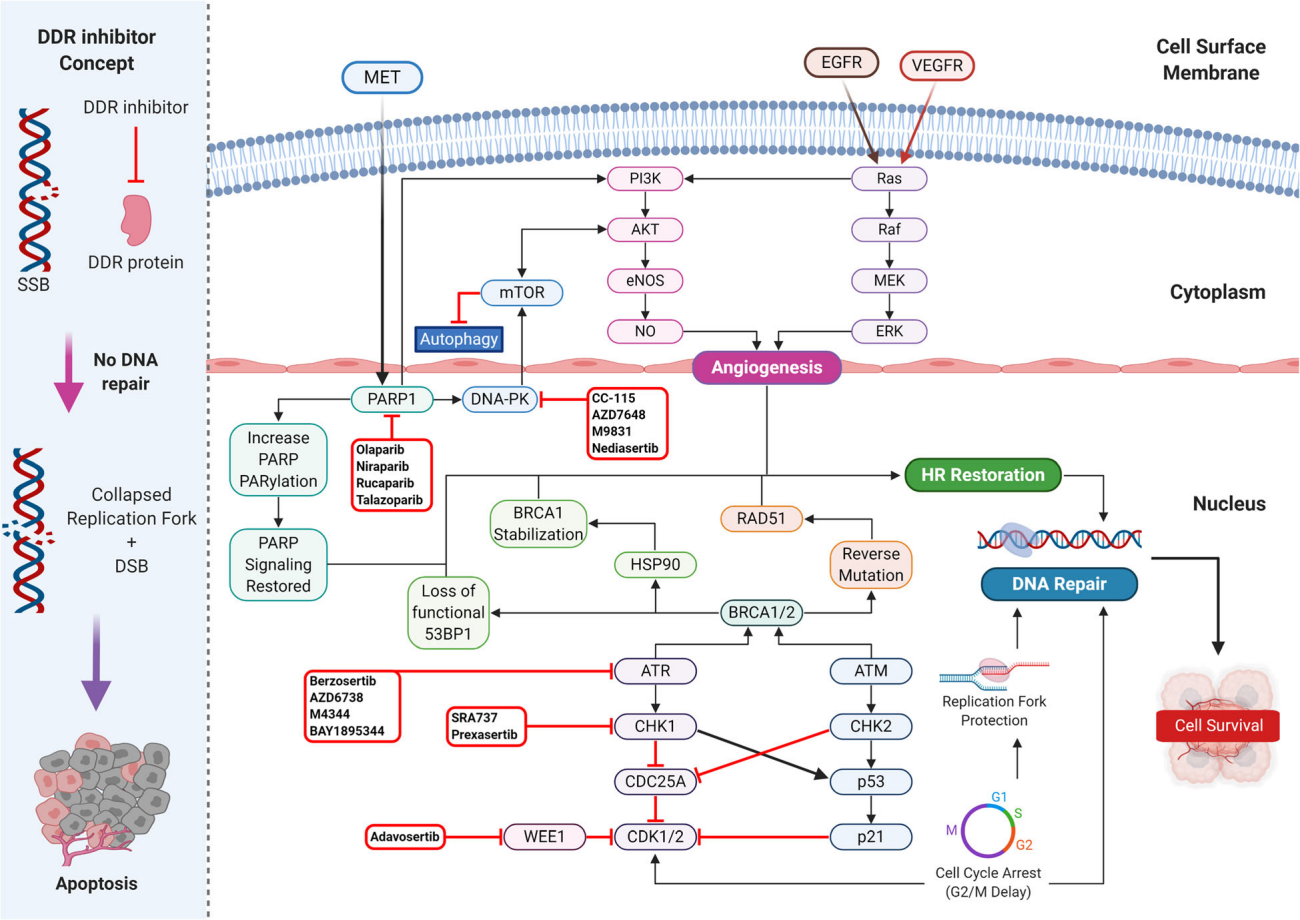
DDR inhibitors mechanisms and targeted pathways in the clinic. (left) The mechanism of DDR inhibitors; DDR inhibitors inhibit the DDR proteins from repairing DNA SSBs, resulting in collapsed replication forks, which leads to DNA DSBs and tumor cell apoptosis. (right) DDR pathways repair DNA through the mitigation of replication stress, therefore the inhibition of these pathways by DDR inhibitors resulted in SSBs and DSBs accumulation. DNA replication is crucial for the DNA repair process, which is associated with replicative stress response and cell cycle regulation. ATM and ATR kinases maintain replication fork stability and regulate the cell cycle control checkpoints together with CHK1/2. The main DDR inhibitors that are currently undergoing clinical trials target the major components of the DDR pathways. The major potential resistance to DDR inhibitors centers around three general mechanisms: replication fork protection, cell cycle arrest, and HR restoration. *DDR* DNA damage response, *SSB* single-strand break, *DSB* double-strand break, *PARP* poly(ADP-ribose) polymerase, *PARG*, poly(ADP-ribose) glycohydrolase, *PARylation* polyADP-ribosylation, *HR* homologous recombination

Synthetic lethality is a phenomenon whereby the concurrent disruption of multiple genes results in cell death, while a disruption in an individual gene is compatible with cell survival (Fig. 1) [9]. Based on targeted therapies acting on the different types of genes driving cancers, synthetic lethality is classified into synthetic sickness lethality and synthetic dosage lethality. In addition, conditional synthetic lethality is regarded as a special type of synthetic lethal interaction. The definition and application of each type of synthetic lethality are summarized in Table 1. There are multiple factors that affect the mechanism of synthetic lethality, including the tumor microenvironment, metabolic pathway, cell cycle control, epigenetic regulation, and the DNA damage response pathway. The major pathways and mechanisms of synthetic lethality are portrayed in Fig. 2.

**Table 1:** Synthetic lethality definition and application

Type		Definition	Application	Reference
Synthetic lethality	Synthetic sickness lethality	The situation in which either of genes in a pair can be excluded without affecting cell viability, while the disruption in both genes concurrently results in cell death. It can be used to target cancers drove by tumor suppressor, which is genetically inactivated by mutation, resulting in the loss of the function of the protein.	PARP shares a synthetic lethal relationship with BRAC1/2, both of which are key in DNA double-strand break repair. BRAC1/2 complete loss of function leaves cells extremely sensitive to PARP inhibitors, thereby presenting a therapeutic opportunity.	[14, 17]
			PI5P4K kinases are essential for cell growth in the absence of p53. Cancer cells with the overexpression of the PI5P4Kβ gene and p53 deficiency lead to senescence, due to the growth phenotype being accompanied by enhanced levels of reactive oxygen species.	[18]
	Synthetic dosage lethality	Overexpression of one gene combined with the loss of function in another gene results in cell death, which can be used for targeting cancer cells with over-expressed, undruggable oncogenes.	MAD2 shares synthetic dosage lethal interaction with PP2A. With the overexpression of MAD2, PP2A inhibition results in lethality in several tumors, including liver cancer, lung cancer, and malignant lymphoma.	[19]
			Histone deacetylases are necessary for tumors with the overexpression of TDP1 and the inhibition of PLK1 in CKS1B over-expressed cells causes cell death.	[20]
Conditional synthetic lethality		Conditional-dependent genetic interactions that depend on synthetic lethal interactions and the genetic background or environment and may account for the variation in synthetic lethal effects observed in different tumor cells.	Cancer cells are usually accompanied with improperly folded proteins, leading proteotoxic stress and the need to increase proteasomal degradation and protein folding capacity for survival, via heat shock proteins upregulation, such as HSF1, HSP70, and HSP90. Interestingly, cancers bearing specific BRAF or EGFR mutations have an increased sensitivity to HSP90 inhibitors.	[21]

Researchers have been assessing the potential of microenvironment-linked synthetic lethality by regulating cellular stress levels. Tumor cells generally suffer more from extreme reactive oxygen species (ROS) or oxidative stress levels, when compared with normal cells, and depends on oxidized nucleotides elimination to survive [22]. Oxidative stress in cancer cells is regulated by the stress-induced transcription factor nuclear factor erythroid 2–related factor 2 (NRF2) [23]. It has been reported that triptolide, a potent NRF2 inhibitor that disrupts glutathione metabolism, have selective cytotoxicity in IDH1-mutated cancer cells [24]. IDH1-mutated cells are dependent on NRF2-guided glutathione synthesis and exhibit increased ROS levels. Therefore, NRF2 inhibition established a synthetic lethal interaction with ROS generated from the mutated IDH1 neomorphic activity. Moreover, proteotoxic stress has been exploited to target tumor cells using synthetic lethality. The combination of heat shock protein 90 (HSP90) and glutaminase inhibitors induces proteotoxic stress and selectively triggers the death of TSC1/2-deficient cells [25]. It has been reported that the major oncogenic pathways have numerous mutated metabolic enzymes [26]. For instance, glycolysis is promoted by RAS or MYC but is inhibited by p53 [27]. AMPK-related kinase 5 shares a synthetic lethal interaction with MYC, as its inhibition leads to ATP reduction and pro-apoptotic response stimulation in cancer cells with MYC overexpression [28]. Thus, metabolism dysregulation is both the cause and the consequence of tumorigenesis [29].

Cell cycle control is a promising method for cancer treatment and synthetic lethality may offer a new direction to inhibit cancer cells directly by specificity utilizing cell cycle control [30]. Synthetic lethal interaction has been observed between Rb and E2F family transcription factors, both of which regulate cellular proliferation by G1 checkpoint restriction. Large-scale short hairpin RNA (shRNA) screens demonstrated that Rb-defective tumor cells rely on the E2F transcription factor family such as E2F3 [31]. In addition, gene encoding protein mutations lead to the loss of chromatin remodeling-associated gene function that is common in malignant tumors [32]. Various studies in yeast genetic interaction network demonstrate that a variety of synthetic lethal interactions among gene pairs in the DDR pathway prevents harmful DNA damage [5, 33]. Researchers are currently exploring various synthetic lethal interactions in the DDR pathway, which will be discussed later in the review. The identified key genes targeted by synthetic lethality approaches are summarized in Table 2.

**Table 2:** Identified key genes in synthetic lethality preclinical studies

Gene	Chromosome	Cellular process and mechanism	In vitro	In vivo	Cancer type	Reference
*ARID1A*	1p36.11	Target SWI/SNF complexes, which regulate chromatin remodeling. SWI/SNF complexes are involved in controlling the cell cycle, DNA replication, and repairing DNA damage.	H1299, H2023, H2030	Smarca4-deficient genetically engineered mouse	Lung cancer	[34]
*ATM*	11q22.3	Activates cell cycle checkpoints; recognizes damaged DNA and triggers ATM-mediated DNA damage response pathway to repair damaged DNA strands.	KC (850, 6059, 8878), AKC (995, 5615, 5980, 5982)	AKC, KC, and SCID mouse	Pancreatic cancer	[35]
*ATR*	3q23	Cell cycle checkpoint signaling activation upon DNA stress and triggers ATR-mediated DNA damage sensing.	Human-derived CLL and Mec1 cell line	Primary CLL xenograft mouse	Leukemia	[36, 37]
*BRCA1*	17q21.31	Repair DNA double-strand breaks via ubiquitination, transcriptional regulation, and homologous recombination.	A2780, HEK293, SUM149PT	N/A	Ovarian cancer	[38]
*BRCA2*	13q13.1	Repair DNA double-strand breaks via ubiquitination, transcriptional regulation, and homologous recombination.	PL2F7, Y3308Y	BRCA-deficient mouse	N/A	[39]
*CDC6*	17q21.2	Initiation of DNA replication; regulates cell cycle.	HCT-116, HKE-3	KRAS-induced lung cancer mouse	Lung cancer	[40, 41]
*CDK1*	10q21.2	Regulate cell cycle (G1/S and G2/M phase transitions).	LIM1215, SW48	KRAS-mutated mouse	N/A	[42]
*CDK2*	12q13.2	Regulate cell cycle (G1/S phase transition).	HACAT	N/A	N/A	[43, 44]
*CDK17*	12q23.1	Serine-threonine protein kinase; regulate G2/M phase transition.	HeLa, K562, MCF10A, MDA-MB-231, RPE1	N/A	Breast cancer	[45]
*CHEK1*	11q24.2	Serine-threonine protein kinase; triggers cell cycle arrest in response to DNA damage; integrate signals from *ATR* and *ATM*; phosphorylation of *CDC25A* to delay cell cycle progression following DNA double-strand breaks.	PEO14, PEO23, SKOV3	SKOV3 xenograft mouse	Ovarian cancer	[46]
*CHEK2*	22q12.1	Serine-threonine protein kinase; triggers cell cycle arrest in response to DNA damage; integrate signals from *ATR* and *ATM*; phosphorylation of *CDC25A* to delay cell cycle progression following DNA double-strand breaks.	Cal27, HN30, HN31, SCC61, UMSCC17A	N/A	Head and neck cancer	[47]
*GATA2*	3q21.3	Zinc-finger transcription factor; regulate transcription genes.	A549, H226, HL7702	A549 xenograft mouse	Lung cancer	[48]
*KRAS*	12p12.1	Transcriptional activator that regulates endothelial cells endothelin-1 gene expression.	A549, H441	A549 xenograft mouse	Lung cancer	[49, 50]
*MRE11*	11q21	MRN complex component; DNA double-strand breaks repair via nonhomologous end-joining and homologous recombination activation in ATM-mediated checkpoint.	V-C8	N/A	N/A	[51]
*MYC*	8q24.21	Regulate cell cycle progression, transcription, and apoptosis.	Kelly, BE-2C, NLF, SK-N-AS, SHEP, MYCN-ER	BALB/c nude mouse	Neuroblastoma	[52, 53]
*NBN*	8q21.3	MRN complex component; DNA double-strand breaks repair via nonhomologous end-joining and homologous recombination activation in ATM-mediated checkpoint.	B220, Gr-1, Mac-1	Nbn-mutated mouse	Leukemia	[54]
*PAK3*	Xq23	Serine-threonine protein kinase; regulates cell cycle, cell migration, and apoptosis.	CaSki, HeLa, HFK, SiHa	N/A	Cervical cancer	[55]
*PARP1*	1q41.42	Regulate cell proliferation and differentiation; repair DNA single- and double-strand breaks.	DLD-1, HEK293FT, KB1P-G3, KB2P, SUM149PT, U2OS	BRCA2-mutated mouse	Breast and ovarian cancer	[56]
*PLK1*	16p12.2	Serine-threonine protein kinase; regulate cell proliferation and apoptosis; triggers G2/M transition.	A549, H441, H522, T29	BALB/c and C57BL/6 nude mouse	Lung cancer	[57]
*RAD50*	5q31.1	MRN complex component; DNA double-strand breaks repair via nonhomologous end-joining and homologous recombination activation in ATM-mediated checkpoint.	D1241, L1240, Q1262, WT	N/A	Metastatic small cell cancer	[58]
*RAD51*	15q15.1	Repair DNA double-strand breaks via homologous recombination.	HeLa, K562, M059, U2OS	N/A	N/A	[59]
*TP53*	17p13.1	Major tumor suppressor; regulate cell cycle, senescence, and apoptosis.	C4-2, LNCaP, U2OS	NSG mouse	Prostate cancer	[60, 61]
*53BP1*	15q15.3	Repair DNA double-strand breaks by promoting non-homologous end-joining pathways while limiting homologous recombination.	DOHH2, G452, HCC1187 OCI-LY (1, 8, 19), SUDHL-6, U2932, VAL	NOD, NSG, and SCID mouse	Lymphoma	[62, 63]
*WEE1*	11p15.4	Serine-threonine protein kinase; regulates G2/M checkpoint via CDC2 inhibition.	MCF7, MDA-MB-231, T-47D, Zr-75-1	Breast cancer xenograft NSG mouse	Breast cancer	[64]

Recently, a new strategy using senolytic agents to induce synthetic lethal interactions have shown great promise in cancer treatment. Cancer therapy can induce tumor cell senescence that negatively affects the tumor microenvironment due to the secreted factors, including various growth factors, chemokines, cytokines, and matrix remodeling enzymes [65]. Senescent cells have stable cell cycle arrest with changes in cell chromatin structure, metabolism, and morphology. It has been reported that tumor development can be inhibited by senescence-associated cell cycle arrest. However, various factors secreted from senescent cells can negatively impact the tumor microenvironment by promoting tumor progression or inducing immune-mediated senescent cell clearance [66]. Mice bearing treatment-induced senescence tumors have extended survival after the elimination of senescent cells, whereas mice bearing senescence-resistant tumors do not [67]. It has been demonstrated that the synthetic lethal approach can be used to treat breast and ovarian cancer cells by inducing senescence with PARP inhibitors and senolytic agents. PARP inhibitor-induced senescence leads to the sensitivity of breast and ovarian cancer cells to second-phase synthetic lethal interaction by using senolytic agents to target the senescence state, increasing the combination therapy efficacy in the xenografted breast (MDA-MB-231) and ovarian (OV4453, OV1946) cancer models [68]. Senolytic agent treatment combined with PARP inhibitors has proven to be effective in preclinical studies, thus the combination of these synthetic lethal interactions could be further explored to limit drug resistance in the clinics.

## Identification of new synthetic lethal interactions

**Fig. 3:**
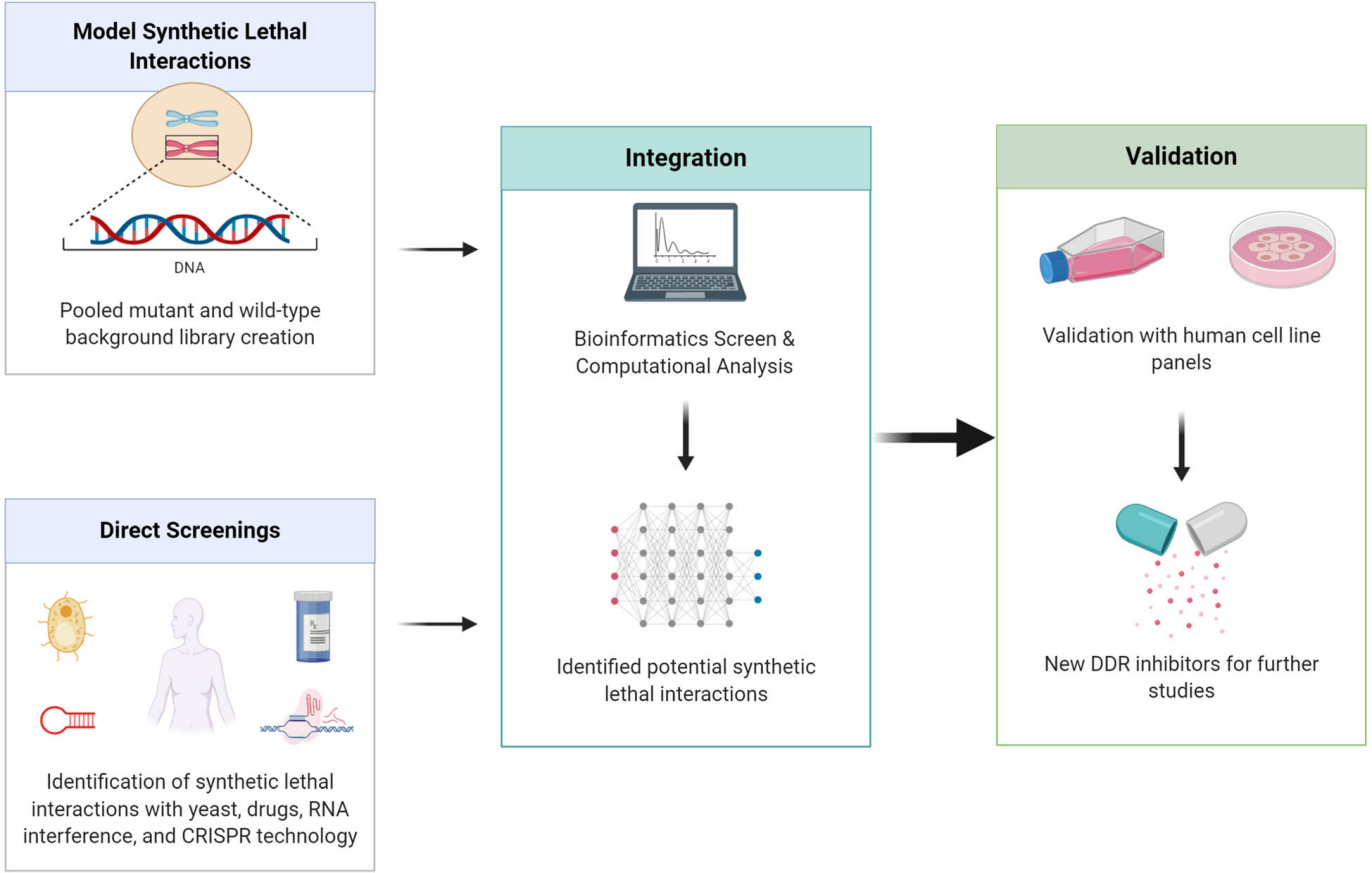
Methods for the identification of synthetic lethal interactions. The potential synthetic lethal interaction data are derived from model organisms (data from BioGRID or TheCellMap), direct screening methods (yeast, drugs, RNA interference, and CRISPR technology), and computational analysis (bioinformatics screen). The discovered synthetic lethal candidate was validated with human cell line panels to determine if the synthetic lethal interaction is limited to specific cell lines or preserved across cell lines

Due to thousands of mutated genes in various cancers, identifying and validating potential synthetic lethal partner genes in various conditions remain a challenge. The five most common synthetic lethal screening approaches include yeast screens, drug screens, RNA interference (RNAi) screens, clustered regularly interspaced short palindromic repeats (CRISPR) screens, and bioinformatics screens (Fig. 3).

### Yeast screens

Model organisms, such as yeast, were used as initial screens to identify synthetic lethality-based anticancer therapeutic targets [69]. Large-scale development of genetic interaction quantification and double mutants is facilitated by high-throughput yeast mating strategies, such as diploid-based synthetic lethality analysis with microarrays and synthetic genetic array [70, 71]. Costanzo et al. [33] generated a comprehensive *Saccharomyces cerevisiae* genetic interaction network and identified over 500,000 synthetic sickness and synthetic lethal interactions. These genetic interaction profiles allow the assembly of the cell function hierarchical model, including modules that correspond to protein pathways, cellular compartments, and biological processes, and provides a clear context for synthetic lethal genetic networks in cancer cell lines [72]. Even though a high-throughput refinement can greatly improve the efficiency of yeast screens, attempts to map synthetic lethal partner genes onto human orthologues still remains a challenge [73].

### Drug screens

Drug screens determine the synthetic lethal interactions between drugs and genes by utilizing the library of drugs on various cell lines with specific mutations. Minor progress has been made via high-throughput drug screens in several tumors including cyclin-dependent kinase 1/2 (CDK1/2)-, polo-like kinase 1 (PLK1)-, and WEE1-mutated cancers. Drug screens are mainly based on both mutation data and high-throughput drug screening data to identify sensitive mutations that may constitute synthetic lethal interactions [74]. Notably, identifying the synthetic lethal interactions through drug screens are reciprocal. Screening drug libraries in cancer with specific mutations and loss-of-function genetic screening of cells treated with selective drugs via RNAi or CRISPR screens can both determine synthetic lethal interactions. In addition, drug dosage also influences the specificity of identifying potential synthetic lethal interactions. If a new gene-drug synthetic lethal interaction is identified, the preclinical validation and clinical trials would be easily performed. However, due to the inadequate target inhibition and possible side effects of the agent, the efficacy and specificity of target drug inhibition are generally lower than genetic knockdown [6].

### RNA interference screens

The development of RNAi screens that can precisely knockdown mRNAs by introducing exogenous small interfering RNA (siRNA) sequences had made genome-wide studies in human cells possible [75]. RNAi screens target post-transcriptional genes by promoting precise mRNAs degradation, which can be divided into short-hairpin and short-interfering screens. RNAi screening technology allows the identification of new genes and genetic networks involved in various biological processes, including protein or organelle function and localization, assays related to cell viability and signal transduction, host cells response to pathogens, and drug resistance [76]. However, RNAi screens are highly prone to off-target effects that will significantly increase treatment risks, thus limiting its clinical application [77]. The consolidation of bioinformatics tools and algorithms improves library generation while reducing off-target effects and increasing the on-target robustness of RNAi sequences to enhance RNAi reagent specificity [78]. Nevertheless, the human cell culture system and RNAi library gene specificity still need to be improved to be used in human RNAi screening.

### CRISPR screens

CRISPR is a compelling technology used to investigate various biological processes such as synergistic and synthetic lethal interactions. It is a scalable genome-editing technology that is highly efficient and specific which surpasses RNAi-based reagents and can be used in high-throughput screens by the utilization of multiple related approaches to discover novel drug targets [79, 80]. The CRISPR-Cas9 system usage in human cells is based on the genome-editing technology development that pairs sequence-specific gRNAs with bacterial Cas9 expression, whereby the enzyme was guided to excise specific DNA fragments of the human genome. Such an approach can be utilized in the human cells to study the systemic effects of the loss of each gene in the genome [81]. The rapid advance in genetic tools and modifications, coupled with recent studies of the technology, provides an insight into the mechanism of CRISPR-Cas9 that will enhance target discovery approaches in the future.

### Bioinformatics screens

Multiple studies have used bioinformatic screening to identify synthetic lethal pathway that can theoretically be used to monitor disease progression [82]. The predictions are produced by scoring gene pair interaction by using expression profiles data, screening results for shRNA, and copy number data [83]. Since the human genome-wide evaluation of synthetic lethal interactions is still impractical, computational data transfer of model organism interactions, ranging from yeast to humans, became the study trend [84, 85]. New innovative programs and computational methods are now being developed to enable the rapid analysis of data sets for gene expression. Despite the limitations of expression profiling, the assimilation of computational methods with other types of data still remains as one of the leading molecular techniques for the prognosis and prediction of synthetic lethal interactions [86].

## Preclinic and clinical development of synthetic lethality

Clinically, the treatment strategy based on synthetic lethality has three major benefits: (1) a synthetic lethality-based treatment strategy could be utilized against the majority of cancer mutations, (2) synthetic lethality allows simple identification of treatment-responding patients due to its selective nature of specific cancer cell genetic mutation, and (3) combination therapy could increase the efficacy of chemotherapeutic drugs, thus allowing for lower dosage and avoiding adverse effects. Currently, the FDA has approved four PARP inhibitors for clinical anticancer therapy, including olaparib, niraparib, rucaparib, and talazoparib. The recent clinical trials for synthetic lethality treatment of tumors, including PARP inhibitors, non-PARP DDR inhibitors, and combination therapies, are summarized in Table 3.

**Table 3:** Recent clinical trials for PARP, ATR, DNA-PK, WEE1, and CHK1 inhibitors: monotherapy and combination therapy

Target	Agent	Intervention	Cancer Type	Phase	ClinicalTrials.gov identifier
PARP	Olaparib	Olaparib	Breast and ovarian cancer	IV	NCT04330040
		Olaparib + Paclitaxel + Durvalumab	Advanced gastric cancer	II	NCT03579784
		Olaparib + Abiraterone	Prostate cancer	III	NCT03732820
		Olaparib + Durvalumab	Bladder cancer	II	NCT03534492
		Olaparib + Temozolomide	Colorectal cancer	II	NCT04166435
	Niraparib	Niraparib	Pancreatic cancer	II	NCT03601923
		Niraparib + Osimertinib	Lung cancer	I	NCT03891615
		Niraparib + Dostarlimab	Ovarian cancer	III	NCT03602859
		Niraparib + MGD013	Gastric and gastroesophageal junction cancer	I	NCT04178460
		Niraparib + Dostarlimab	Cervix cancer	II	NCT04068753
	Rucaparib	Rucaparib	Endometrial cancer	II	NCT03617679
		Rucaparib + Nivolumab	Biliary tract cancer	II	NCT03639935
		Rucaparib + Radiotherapy	Breast cancer	I	NCT03542175
		Rucaparib + Copanlisib	Prostate cancer	I	NCT04253262
		Rucacparib + Enzalutamide + Abiraterone	Prostate cancer	I	NCT04179396
	Talazoparib	Talazoparib	Leukemia	I	NCT03974217
		Talazoparib + Avelumab	Breast cancer	I	NCT03964532
		Talazoparib + Radiotherapy	Gynecologic cancer	I	NCT03968406
		Talazoparib + ASTX727	Breast cancer	I	NCT04134884
		Talazoparib + Avelumab	Lung cancer	II	NCT04173507
		Talazoparib + Axitinib	Kidney cancer	I/II	NCT04337970
		Talazoparib + Atezolizumab	Lung cancer	II	NCT04334941
		Talazoparib + Gedatolisib	Breast cancer	II	NCT03911973
ATR	Berzosertib (M6620)	Berzosertib + Radiotherapy	Lung cancer	I	NCT02589522
		Berzosertib + Radiotherapy	Breast cancer	I	NCT04052555
		Berzosertib + Topotecan	Lung cancer	I/II	NCT02487095
		Berzosertib + Topotecan Hydrochloride	Lung cancer	II	NCT03896503
		Berzosertib + Carboplatin + Docetaxel	Prostate cancer	II	NCT03517969
	AZD6738	AZD6738 + Radiotherapy	Advanced solid tumors	I	NCT02223923
		AZD6738 + Olaparib	Gynecologic cancer	II	NCT04065269
		AZD6738 + Olaparib + Durvalumab	Breast cancer	II	NCT03740893
		AZD6738 + Acalabrutinib	Chronic lymphocytic leukemia	I/II	NCT03328273
		AZD6738 + Durvalumab	Biliary tract cancer	II	NCT04298008
	BAY1895344	BAY1895344	Advanced solid tumors	I	NCT03188965
		BAY1895344 + Pembrolizumab	Advanced solid tumors	I	NCT04095273
		BAY1895344 + Niraparib	Ovarian cancer	I	NCT04267939
	M4344	M4344 + Niraparib	Ovarian cancer	I	NCT04149145
		M4344 + Carboplatin	Advanced solid tumors	I	NCT02278250
DNA-PK	CC-115	CC-115	Advanced solid tumors	I	NCT01353625
		CC-115 + Enzalutamide	Prostate cancer	I	NCT02833883
	AZD7648	AZD7648 + Olaparib + Pegylated Liposomal Doxorubicin	Advanced solid tumors	I/II	NCT03907969
	M9831 (VX-984)	M9831	Advanced solid tumors	I	NCT02644278
	Nedisertib (M3814)	Nedisertib + Pegylated Liposomal Doxorubicin Hydrochloride	Ovarian cancer	I	NCT04092270
		Nedisertib + Avelumab + Radiotherapy	Hepatobiliary cancer	I/II	NCT04068194
		Nedisertib + Avelumab + Radiotherapy	Advanced solid tumors	I	NCT03724890
WEE1	Adavosertib (AZD1775)	Adavosertib	Advanced solid tumors	I	NCT01748825
		Adavosertib	Advanced solid tumors	II	NCT03253679 / NCT03284385
		Adavosertib + Gemcitabine + Cisplatin + Carboplatin	Advanced solid tumors	I	NCT00648648
		Adavosertib + Olaparib	Ovarian, primary peritoneal, and fallopian tube cancer	II	NCT03579316
		Adavosertib + Olaparib + AZD6738	Breast cancer	II	NCT03330847
		Adavosertib + Irinotecan	Advanced solid tumors	I/II	NCT02095132
		Adavosertib + Cisplatin + Radiotherapy	Cervical, vaginal, and uterine cancer	I	NCT03345784
		Adavosertib + Temozolomide + Radiotherapy	Glioblastoma	I	NCT01849146
CHK1	SRA737	SRA737	Advanced solid tumors	I/II	NCT02797964
		SRA737 + Gemcitabine + Cisplatin	Advanced solid tumors	I/II	NCT02797977
	Prexasertib (LY2606368)	Prexasertib	Advanced solid tumors	I	NCT01115790
		Prexasertib	Lung cancer	II	NCT02735980
		Prexasertib	Breast, ovarian, and prostate cancer	II	NCT02203513
		Prexasertib + Cisplatin + Cetuximab + Radiotherapy	Head and neck cancer	I	NCT02555644

### PARP inhibitors in clinical practice

The synthetic lethal interaction among PARP and BRCA1/2 was discovered in 2005 [10, 11]. PARP-1 is a DNA repair protein that regulates cell proliferation and differentiation by repairing DNA single-strand break (SSB) and double-strand break (DSB). The inhibition of PARP1 DNA damage repair by PARP inhibitors leads to deleterious mutation accumulation, resulting in genetic instability and cell death [56]. PARP inhibitors initially entered the clinical trials as combination therapy of full-dose temozolomide, a DNA alkylating agent, and low-dose rucaparib [87]. A phase 1 olaparib clinical trial, which involved patients with BRCA1/2 mutations, reported that 63% of the patients who received olaparib exhibited clinical benefit with minimal side effects than those of conventional chemotherapy regimens [16]. Subsequently, phase 2 and 3 clinical trials, which included patients with BRCA1/2-mutated breast, ovarian, pancreatic, and prostate cancers, demonstrated the clinical benefit offered by olaparib [88–92], thus providing sufficient evidence for the FDA to approve olaparib as a treatment for advanced ovarian cancer patients [93, 94].

The FDA approved niraparib as maintenance therapy for the primary peritoneal, fallopian tube, or ovarian cancer patients [95]. The phase 1 dose-escalation trial with 100 advanced solid tumor patients assessed the safety, efficacy, and tolerability of niraparib monotherapy. The results from the pharmacodynamic analysis demonstrated that anticancer activities were recorded at doses above 60 mg/day and PARP inhibition over 50% at doses above 80 mg/day [96]. The FDA approval of niraparib was based on the evidence from the phase 3 NOVA trial with 553 platinum-sensitive recurrent ovarian cancer patients. Based on the phase 1 trial results [96], patients in the NOVA trial received niraparib 300 mg/day for 28-day cycles. Patients that received niraparib had a significantly longer progression-free survival (PFS) despite the germline BRCA mutation status (21.0 vs. 5.5 months for BRCA-mutated group; 9.3 vs. 3.9 months for non-BRCA-mutated group; hazard ratio 0.45, *P* < 0.001 for all groups) [97]. The most common niraparib adverse effects are thrombocytopenia, neutropenia, nausea, fatigue, and anemia, which could be controlled with dosage modifications [96, 97].

In addition, rucaparib has demonstrated an increase in maintenance setting PFS [98]. In phase 2 ARIEL trial assessing rucaparib, 206 high-grade ovarian carcinoma patients were categorized into three different groups based on the features of the tumor genome. The BRCA-mutant group has the best PFS, followed by the high chromosomal loss of heterozygosity group with a slightly more benefit over the low chromosomal loss of heterozygosity group (median PFS 12.8 vs. 5.7 vs. 5.2 months, respectively) [99]. Subsequently, the phase 3 ARIEL trial with 564 platinum-sensitive ovarian cancer patients confirmed that rucaparib significantly improved PFS (16.6 vs. 5.4 months for rucaparib and placebo group, respectively) [100]. Furthermore, ARIEL 3 demonstrated that PARP inhibitors could be used in platinum-sensitive ovarian cancer patients with previous platinum-based chemotherapy treatment. Therefore, according to the clinical trial results, rucaparib was approved by the FDA to treat BRCA1/2-mutated advanced ovarian cancer patients that received two or more chemotherapies [99, 100].

Talazoparib recently received FDA approval to treat HER2-negative, BRCA-mutated advanced breast cancer patients [101]. The phase 1 trial, consisting of 18 BRCA1/2-mutated advanced breast cancer patients receiving talazoparib monotherapy (1 mg QD), demonstrated 86% clinical benefit rate with 50% response rate at 24 weeks (median PFS 34.6 weeks) [102]. The most frequent side effects of talazoparib include thrombocytopenia, fatigue, and anemia. Phase 2 ABRAZO trial with 84 BRCA1/2-mutated metastatic breast cancer patients receiving talazoparib monotherapy resulted in a 37% response rate for patients who previously received more than two advanced breast cancer cytotoxic regimens without exposure to platinum chemotherapy and 21% response rate for patients who previously are responsive to platinum chemotherapy (median PFS 5.6 vs. 4.0 months, respectively) [103]. Subsequently, the phase 3 EMBRACA trial with 431 BRCA1/2-mutated advanced breast cancer patients, talazoparib monotherapy exhibits significant PFS benefit when compared with standard chemotherapy, with the talazoparib group objective responsive rate significantly higher than the standard chemotherapy group (62.6% vs. 27.2%, respectively; *P* < 0.001) [104]. These results indicate that talazoparib has significant PFS and overall survival improvements, leading to the recent approval of talazoparib by the FDA.

**Fig. 4:**
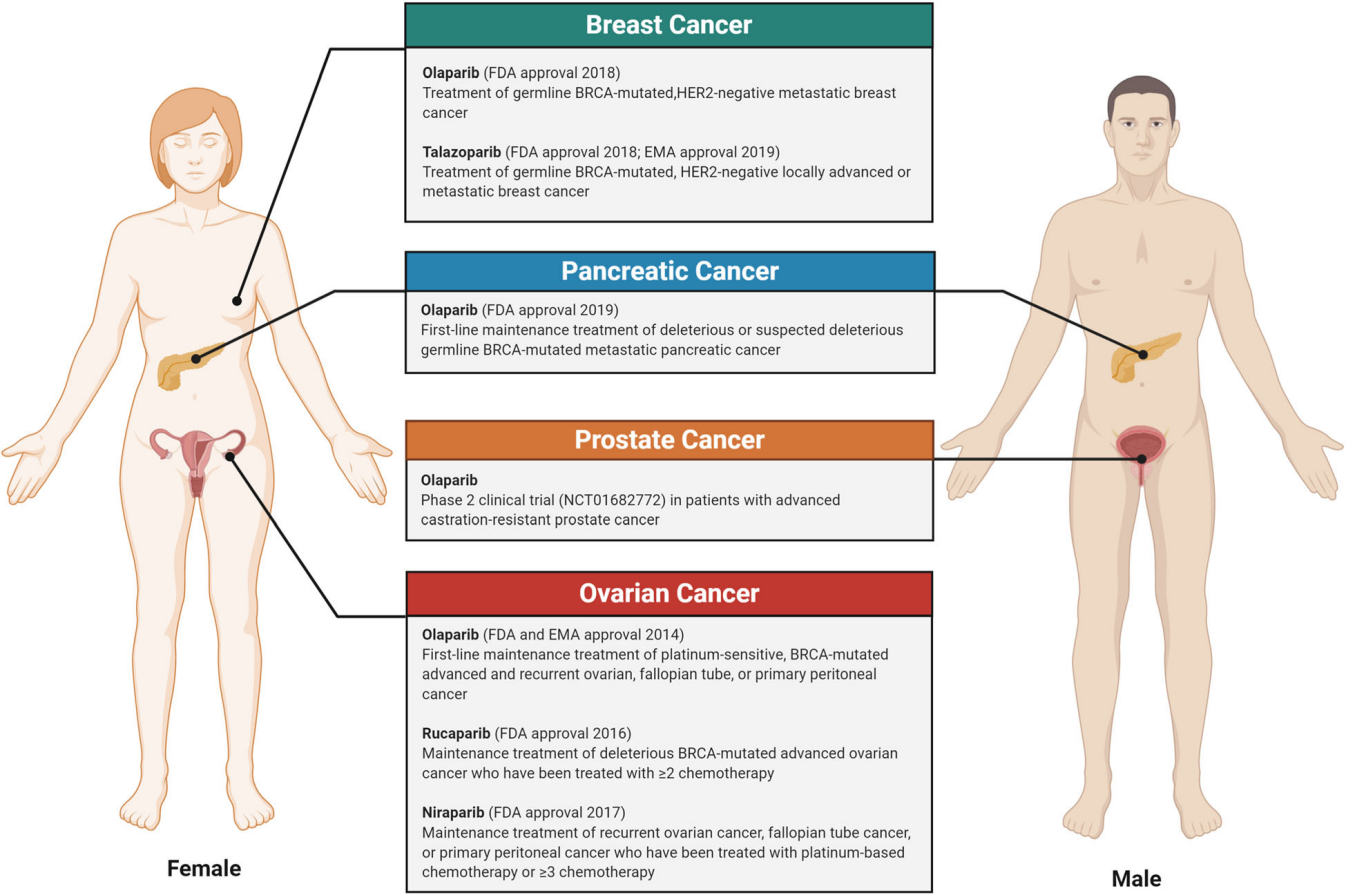
Clinical assessment of PARP inhibitors in cancer therapy. PARP inhibitors based on the concept of synthetic lethality mainly focuses on germline BRCA1/2-mutated tumors. Various PARP inhibitors have been approved by regulatory bodies, such as the United States Food and Drug Administration (FDA) and the European Medicines Agency (EMA), to treat patients with BRCA-mutated breast, pancreatic, and ovarian cancers. Olaparib is currently undergoing phase 2 clinical trial for the treatment of advanced castration-resistant prostate cancer

Although early clinical trials regarding synthetic lethality were focused on PARP inhibitors efficacy in germline-mutated BRCA1/2 cancers, further studies discovered that the responses are not limited to tumors with these mutations [16]. The clinical assessment of PARP inhibitors approved by the FDA is summarized in Fig. 4. In order to increase patients that would benefit from treatments based on synthetic lethality, tremendous efforts are being made to identify mutations that are susceptible to PARP and other DDR inhibitors in the HR pathway.

### Synthetic lethality beyond PARP inhibitors

The revelation of synthetic lethality in various DNA repair pathways expanded DDR and DNA repair-targeting clinical strategies, with various DDR inhibitors in preclinical and clinical development. The encouraging results from PARP inhibitors led to an increasing amount of research focused on targeting other DDR pathway components as synthetic lethal approaches for cancer treatment. The lessons learned from PARP inhibitors and a comprehensive understanding of the DDR pathways are being combined to maximize the potential and clinical success of DDR inhibitors. The inhibition of DDR pathways such as ataxia telangiectasia and Rad3-related (ATR) inhibitors, DNA-dependent protein kinase (DNA-PK) inhibitors, WEE1 inhibitors, and checkpoint kinase 1/2 (CHK1/2) inhibitors have shown promising results clinically.

#### ATR mechanisms and inhibitors

ATR and ATM are one of the primary targets of DDR inhibitors due to its central regulatory function that works through overlapping but distinct pathways to activate DDR [105]. In the initial phase of homologous recombination (HR), DNA end resection leads to stalled replication forks that activates ATR by replication protein A (RPA)-bound ssDNA [106]. RPA-ssDNA is induced through the TOPBP1 complex with Claspin, RHINO, RAD9-RAD1-HUS1 complex, and RAD17-RFC2-5 clamp loader, which activates ATR to RPA-ssDNA via ATR-interacting protein [59]. In contrast, ATM responds to the generated DNA DSB throughout the cell cycle and promotes DSB repair. Following the detection of DNA DSBs, it has been reported that ATM is primarily activated by interacting with NBS1 from the MRN complex [107]. Oncogene activation and loss of G1 checkpoint control that drives cancer cell replication, leading to the cancer cell dependency on the S and G2/M checkpoints and increased replication stress when cancer cells enter the S phase [108]. In addition, chemotherapy, radiotherapy, and cancer-associated inflammation also result in higher replication stress. Therefore, the synthetic lethal interaction can be exploited as the result of increased dependency on the S and G2/M checkpoints mediated by ATR due to the heightened replication stress as the cancer cells enter S phase. Since ATR is crucial in the intra-S and G2/M phase cell cycle checkpoints, ATR inhibitors cause DNA damage accumulation and cell death by selectively targeting tumor cells with DNA damage-induced G1 cell cycle checkpoints defects [109].

Despite the limited data on ATR inhibitor efficacy in the clinics, patients with ATM-mutated cancer that receives an ATR inhibitor shows a complete clinical response [110]. The four ATR inhibitors that are undergoing clinical trials include berzosertib, AZD6738, BAY1895344, and M4344. Berzosertib has been tested in several clinical trials as a monotherapy and combination therapy [111, 112]. Patients receiving berzosertib monotherapy demonstrated fine tolerance and no dose-limiting toxicities. The pharmacodynamic studies of phase 1 clinical trial of berzosertib plus topotecan in 21 patients with advanced solid tumors revealed that ATR inhibition leads to enhanced DNA DSBs, resulting in stabilized or improved symptoms for patients (median PFS 10.2 months; confidence interval 95%, 1.4 to 10.2 months) [112]. In addition, the on-going phase 1 PATRIOT trial with advanced solid tumor patients is assessing the safety and efficacy of AZD6738 monotherapy. Due to the observed bone marrow suppression after continuous dosing beyond the first cycle, various dosing schedules are being examined to improve the long-term tolerability of the patients [113]. Currently, BAY1895344 and M4344 are being tested in phase 1 clinical trial in combination with chemotherapies and as monotherapy for advanced solid tumor patients.

#### DNA-PK mechanisms and inhibitors

It has been reported that DNA-PK suppresses tumor growth by regulating the transcription of cancer-related pathways genes in vitro, in vivo, and ex vivo [114]. It is an essential enzyme in the PI3K-mTOR family that functions in the non-homologous end joining (NHEJ) DNA repair pathway, a predominant DSB DNA repair pathway [110]. DNA-PK comprises of DNA-PKcs, a catalytic subunit, and Ku, which is activated by the binding of Ku to DNA DSBs, leading to major NHEJ protein recruitment such as DNA-PKcs, LIG4, PAXX, XLF, and XRCC4. NHEJ core complex is destabilized upon the autophosphorylation of DNA-PKcs, resulting in Ku inward sliding on the DNA and facilitating the access of ligation enzymes to repair DNA ends [115]. It has been demonstrated that BRCA1 deficiency with genetic or pharmacological inactivation of DNA-PK results in synthetic lethality. BRCA1 deficiency is commonly associated with the decreased expression of key factors in alternative NHEJ and base excision repair (BER), specifically apurinic/apyrimidinic endonuclease 1 (APE1), DNA polymerase b (POLB), and X-ray repair cross-complementing 1 (XRCC1) [116]. Impaired BER and alternative NHEJ repair pathway inhibition results in the decreased expression of these factors, leading to the accumulation of unrepaired SSBs and ensuing DSBs [117]. In addition, cancer cells with overexpressed MYC oncogene are reported to be synthetically lethal to DNA-PK inhibition, supposedly due to increased MYC-driven DNA damage and the cancer cell reliance on the DDR pathways [118].

There are four DNA-PK inhibitors that are undergoing clinical trials, including CC-115, AZD7648, M9831, and nedisertib. CC-115, a small-molecule mTOR and DNA-PK inhibitor [119], is undergoing phase 1 clinical trial which includes 44 advanced solid or hematologic malignancy patients receiving CC-115 monotherapy. The most common adverse event for CC-115 is hyperglycemia, which is associated with the inhibition of mTOR complex 1 and 2 [120]. In addition, AZD7648 is a toxic and highly selective DNA-PK inhibitor that has been shown to induce genomic instability, inhibit cell growth, and apoptosis in ATM-deficient cells, when used in combination with olaparib [121]. Based on the AZD7648 preclinical data, phase 1/2a clinical trial consisting of 234 patients receiving AZD7648 alone and in combination with olaparib has recently been initiated [122]. Furthermore, M9831 and nedisertib are currently in phase 1 clinical trials under monotherapy and in combination with chemotherapies for advanced solid tumor patients.

#### WEE1 mechanisms and inhibitors

WEE1 protein kinase has been reported to inhibit CDK 1 and 2, which activates G2/M cell cycle checkpoint, resulting in temporary cell cycle arrest and DNA damage repair. Hence, WEE1 inhibitors prevent the activation of G2/M cell cycle checkpoint, resulting in the loss of genomic integrity due to increased replication stress [123]. The inhibition of WEE1 protein kinase produces replication-dependent intracellular DNA damage due to abnormal DNA replication through CDK 2 inhibition [64]. In addition, p53 regulates the G1 cell cycle checkpoint, which leads to an increased reliance on the G2 cell cycle checkpoint of p53-deficient cells. Thus, WEE1 inhibitors are used to target p53-deficient tumors. Several preclinical studies confirmed that WEE1 inhibitors cause mitotic lethality, making p53-deficient cells sensitive to radiation and DNA damaging agents [124, 125]. For instance, Liang et al. [126] reported that apoptosis induced by accumulated DNA damage increases the sensitivity of cancer cells to WEE1 inhibitors. WEE1 inhibitor can selectively inhibit the proliferation of glioma and hepatocellular carcinoma cells with ATRX mutations, which indicates that the synthetic lethal interaction between ATRX and WEE1 can be applied in an extensive range of tumors. Therefore, researchers are focusing on the synergistic activity of WEE1 inhibitors with different DNA damaging agents.

Adavosertib is the only WEE1 inhibitor that is currently undergoing clinical trials. The preclinical data demonstrate that adavosertib inhibits the G2 cell cycle checkpoint and renders p53-deficient tumor cells vulnerable to radiation and DNA-damaging chemotherapies [127, 128]. The phase 1 clinical trial with 25 refractory solid tumor patients reported that two BRCA-mutated tumor patients in the study have partial response to adavosertib monotherapy [129]. Moreover, another phase 1 clinical trial consisting of 202 advanced solid tumor patients receiving adavosertib in combination with chemotherapies resulted in 53% disease stabilization and 10% partial response [130]. Both of which confirmed the safety and efficacy of adavosertib monotherapy and in combination with chemotherapy. Subsequently, the phase 2 trial with 24 p53-mutated ovarian cancer patients receiving adavosertib plus carboplatin reported that the overall response rate was 43% (confidence interval 95%, 22% to 66%) [131]. The main adverse effects reported by the adavosertib clinical trials were fatigue, nausea, and thrombocytopenia.

#### CHK1 mechanisms and inhibitors

CHK1 and 2 are cell cycle checkpoint kinases in the DDR pathway that are targeted by ATR and ATM, respectively. It prevents cell cycle progression when DNA damage is detected and being repaired [132]. CHK1 kinase has several targets that facilitate the S and G2/M phase cell cycle checkpoint arrest and could phosphorylate and degrade CDC25A, CDC25B, and CDC25C phosphatases at multiple sites, resulting in increased CDK protein phosphorylation and inhibition [133]. Rogers et al. [134] indicated that the silencing of DNA polymerase family B subunits (POLE, POLE2, and POLA) results in an increased sensitivity of colorectal cancer (SW620) and non-small cell lung cancer (A549) cells to CHK1 inhibitors. The depletion of DNA polymerase family B coupled with CHK1 inhibitor leads to increased DNA damage, replication stress, and apoptosis in cancer cells. Furthermore, treatment-induced inhibition of DNA polymerase family B via CD437 or aphidicolin with CHK1 inhibitor synergistically inhibited the proliferation of cancer cells. Moreover, CHK1 has been reported to activate WEE1 kinase and inhibit CDK1 [135]. Hence, CHK1 inhibitors were developed to facilitate DNA-damaged cell progression.

Multiple studies have reported that CHK1 inhibitors are highly synergistic with replication-dependent DNA damage generating drugs, thus combination therapy with such drugs is focused during clinical development [136, 137]. CHK1 inhibitors have been developed and tested with various drugs incessantly due to toxicity concerns. The current ongoing clinical trials for CHK1-selective inhibitors include SRA737 and prexasertib. SRA737 is an oral CHK1 inhibitor that is in phase 1 and 2 clinical trials as a monotherapy and in combination with low-dose gemcitabine for non-Hodgkin’s lymphoma and advanced solid tumor patients [137]. In addition, the second-generation CHK1 inhibitor prexasertib is also being tested in the clinical trials. The phase 1 trial reported that transient neutropenia was frequently observed in patients receiving prexasertib monotherapy [138, 139]. Subsequently, the phase 2 prexasertib trial consisting of 28 high-grade serous ovarian cancer patients demonstrated a 29% partial response (confidence interval 95%, 13% to 49%) for prexasertib monotherapy, with the most common adverse effects being decreased white blood cell count and neutropenia [140].

### Combination therapies

#### DDR inhibitors and DNA-damaging agents

As previously mentioned, PARP inhibitors cause irreparable cytotoxicity by inducing the collapsing/stalling of the DNA replication fork to block cellular replication and inhibiting repair protein recruitment at DNA-damaged sites. PARP inhibitors were initially designed to make tumors vulnerable to DNA-damaging agents such as radiation and chemotherapeutic agents. Indeed, PARP inhibitors succeeded in sensitizing tumor cells to topoisomerase 1 inhibitors (e.g., camptothecin) and radiation [141]. However, it has been reported that the combination of PARP inhibitors with doxorubicin, gemcitabine, and taxan has no significant synergistic effects [142]. Lu et al. [143] indicated that PARP inhibitors determine the efficacy of the combination therapy with the same chemotherapeutic agent. For instance, a randomized phase 2 trial reported that veliparib, a potent PARP inhibitor, has higher PFS and overall survival in patients treated with veliparib combined with carboplatin/paclitaxel compared to patients treated with veliparib combined with temozolomide (median PFS 14.1 vs. 7.4 months and median overall survival 28.3 vs. 19.1 months, respectively) [144]. Each therapeutic agent can act according to the defined molecular mechanism while having a limited effect on other agents in combination therapy. Hence, combination therapies with PARP inhibitors could be utilized based on the dual molecular mechanism in various types of solid tumors. Moreover, DDR inhibitors have been combined with DNA-damaging agents in multiple clinical trials. In order to maximize synergistic anticancer efficacy, a complete understanding of the DNA damage induced by the respective pathways inhibition and various chemotherapies are required. Nonetheless, it is imperative to consider the toxicities resulted from such combinations. The limitations for combination therapies could be overcome by a precise selection of cancer patients with specific phenotypes or genotypes and a thorough analysis of the combination drug administration sequence, to optimize the synergistic effect of the drugs.

#### DDR inhibitors and immune checkpoint inhibitors

Recently, various studies have reported that PARP inhibitors could enhance the efficacy of immune checkpoint inhibitors by alleviating resistance through immune microenvironment modification and inducing cross-presentation [145]. The combination of PARP inhibitors with immune checkpoint inhibitors demonstrated promising results in ovarian cancer treatment. The DNA damage induced by PARP inhibitors enhances immune priming through various molecular mechanisms and upregulates the expression of programmed death-ligand 1 (PD-L1) [146]. Ding et al. [147] showed that olaparib activated the stimulator of interferon genes (STING) pathway and increased the expression of C-X-C motif chemokine ligand 10 (CXCL10), interferon-beta (IFN-β), and PD-L1 in high-grade serous ovarian cancer mice model with p53 and BRCA1 deficiency and c-MYC overexpression. The combination of programmed cell death protein 1 (PD-1) inhibitor with olaparib enhanced the treatment efficacy of olaparib, whereas anti-PD-1 monotherapy shows no effect. Likewise, Shen et al. [148] demonstrated that talazoparib also activated the STING pathway and increased the expression of chemokine ligand 5 (CCL5), CXCL10, and PD-L1. Talazoparib and anti-PD-L1 antibody combination therapy exhibited synergistic activity in the HR proficient ID8 mice model. Furthermore, the phase 1 clinical trial consisting of 9 female patients with women’s cancer (ovarian, endometrial, and breast cancer) receiving a combination of durvalumab (PD-L1 inhibitor), cediranib (VEGFR1-3), and olaparib reported 67% clinical benefit rate (44% partial response and 33% stable disease ≥ 6 months) without dose-limiting toxicities [149]. The phase 2 expansion study is currently being conducted with recurrent ovarian cancer patients.

Various studies have shown that other DDR inhibitors, such as ATR, WEE1, and CHK1 inhibitors, have synergistic effects with immune checkpoint inhibitors. Sheng et al. [150] demonstrated that AZD6738 enhances the efficacy of immune checkpoint inhibitors and radiotherapy in hepatocellular carcinoma. The synergistic antitumor effect of AZD6738 and radioimmunotherapy combination therapy depended on cyclic GMP–AMP synthase (cGAS)/STING signaling pathway activation. AZD6738 increased cell proliferation, infiltration, and interferon-gamma (IFN-γ) production of tumor-infiltrating lymphocyte CD8^+^ T cells, resulting in decreased T cells and tumor-infiltrating lymphocyte Tregs in mice xenografts. Moreover, Patel et al. [151] indicated that adavosertib and ionizing radiation combination therapy enhanced the sensitivity to T-lymphocyte, tumor-specific cytotoxicity, and programmed death-axis immune checkpoint blockade response in various cancers such as melanoma, lung carcinoma, and head and neck carcinoma in vitro and in vivo. The addition of adavosertib after ionizing radiation reversed the G2/M cell cycle checkpoint activation and led to cell death. Adavosertib and ionizing radiation combination therapy promote the accumulation of M-phase DNA damage in cells, resulting in mitotic catastrophe. In addition, Sen et al. [137] reported that SRA737 and anti-PD-L1 combination therapy significantly decreased the population of myeloid-derived suppressor cells and immunosuppressive M2 macrophages and increased the expression of CCL5, CXCL10, and IFN-β, which enhances the anti-cancer immune response in multiple cancer cells.

## Synthetic lethality limitations and drug resistance

**Fig. 5:**
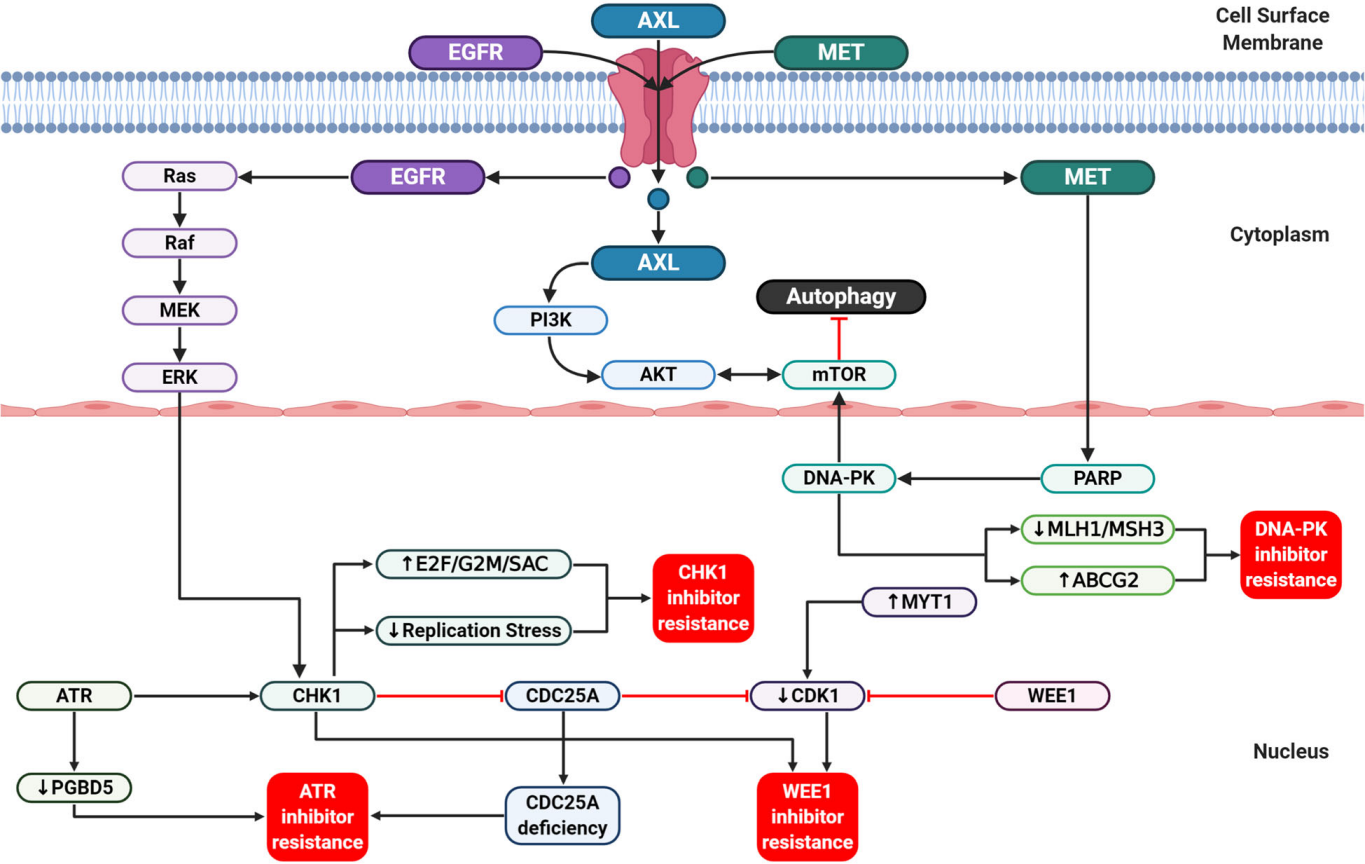
Major DDR inhibitor drug resistance mechanisms. DDR inhibitor resistance can be acquired or inherent. ATR inhibitor resistance is determined by the PGBD5 depletion and CDC25A deficiency. DNA-PK inhibitor resistance is caused by the loss of MLH1/MSH3 and the overexpression of ABCG2. WEE1 inhibitor resistance is induced by AXL overexpression, mTOR signaling, and CHK1 activation; the overexpression of MYT1 levels lowers CDK1 activity and contributes to WEE1 inhibitor resistance. CHK1 inhibitor resistance is associated with increased E2F/G2M/SAC expression and reduced replication stress. *DDR* DNA damage response, *PARP* poly(ADP-ribose) polymerase, *PARG* poly(ADP-ribose) glycohydrolase

There are several limitations in the development of synthetic lethal drugs (Fig. 5). First, cancers generally do not rely on one DNA repair pathway to survive. The DNA repair pathways could overlap and result in the resistance of a synthetically lethal drug [110]. Second, most DNA repair pathways have similar DNA repair proteins instead of independent domains. Thus, the synthetic lethal drugs that inhibit a single DNA repair component may have off-target side effects, in which the shared domain of the targeted repair component of another important protein is also inhibited. Furthermore, these DDR inhibitors may increase the side effects of anticancer drugs and DNA damage on normal tissue, which increases the risk of secondary malignancies. Third, the tumor-specific DNA repair components for various cancer types are not well defined [152]. However, this issue could be overcome as genome-wide sequencing for various tumors are further reported.

### Resistance to PARP inhibitors

Since PARP inhibitors have been used clinically over an extended period of time, it has been reported that cancers resistance to PARP inhibitor is multifactorial and can either be acquired or inherent [153]. Replication stress mitigation, a process which tumor cell stabilizes the replication forks and slow down the cell cycle by various pathways, is crucial in the cancer resistance to PARP inhibitors [154]. The increased protection of the replication fork and the decreased proliferation through mechanisms, such as the dependence on the ATR-CHK1 replication checkpoint pathway and the loss of EZH2 or MLL3/4 for DNA repair, lead to the resistance of PARP inhibitors [155]. Preclinical studies have reported that the mechanisms of PARP inhibitors resistance are associated with HR repair activity restoration. HR repair functions could be restored through indirect mechanisms, such as signals that increase the expression or activity of the HR machinery, and direct mechanisms, including epigenetic, genomic, or post-translational variations in the HR machinery. Secondary mutations across various cancer types were discovered to cause the reversion of DDR protein or gene function, such as BRCA1/2, PALB2, and RAD51C/D, resulting in the restoration of HR repair function and nullifying synthetic lethality [156, 157]. In addition, the loss of 53BP1, resulting in DNA repair balance shifting from NHEJ to HR, is associated with PARP inhibitors resistance [62]. Most PARP inhibitor resistance biomarkers do not rely on one DDR pathway. For instance, cancer cell resistance to PARP inhibitors can emerge through the loss or mutations of PARP1 and lead to a decrease of PARP inhibitor binding. The insufficient PAR glycohydrolase (PARG) activity raises the auto-PARylation of PARP1, resulting in the restoration of PARP signaling which releases PARP1 from the DNA and decreases the DNA damage efficacy of PARP inhibitors [56, 158].

### Resistance to ATR inhibitors

Thus far, ATR inhibitor resistance has not been reported in clinical studies. However, several preclinical studies have identified the mechanism of ATR inhibitor resistance in vitro. Henssen et al. [159] recently reported that endogenous PGBD5 depletion is associated with AZD6738 resistance in human tumor cells. AZD6738 causes unrepaired DNA damage to be accumulated in PGBD5-expressing cells, resulting in G1-phase and dividing cell apoptosis. Ruiz et al. [160] indicated that CDC25A determines ATR inhibitor sensitivity. CDC25A-deficient cells do not enter mitosis prematurely, which leads to the resistance of high dose ATR inhibitors. However, WEE1 inhibitors force CDC25A-deficient cells to enter mitosis and could restore the cytotoxicity of ATR inhibitors.

### Resistance to DNA-PK inhibitors

The molecular mechanisms of DNA-PK inhibitor resistance have been explored by several studies. It has been reported that KU60648, a DNA-PK inhibitor, is sensitive to the loss of MMR protein MSH3 in MLH1-deficient cells [161]. Hinrichsen et al. [162] discovered that MLH1-deficient cells are sensitive to KU60648 compared with overexpressed MLH1 or MMR-proficient cells due to the decreased DSB repair capacity. Moreover, Beebe et al. [163] indicated that the overexpression of ATP-binding cassette G2 (ABCG2), an ATP-binding cassette transporter superfamily member, increases CC-115 resistance. Since CC-115 is a substrate of ABCG2, its potency is affected by ABCG2 expression; thereby the inhibition of ABCG2 via small molecular inhibitors, tumor cells will be sensitive to CC-115.

### Resistance to WEE1 inhibitors

Although adavosertib has shown promising results in the clinical trials, drug resistance is inevitable. Sen et al. [164] demonstrated that AXL and phosphorylated ribosomal S6 (pS6) induces WEE1 inhibitor resistance through downstream mTOR signaling and the activation of CHK1. Adavosertib-resistant cells exhibit high AXL and pS6 expression level, thus WEE1 inhibitors combined with AXL or mTOR inhibitors could overcome adavosertib resistance. In addition, AXL activates the ERK pathway to recruit and activate CHK1. Adavosertib-resistant cells have overexpressed AXL, MET, and pS6 levels that could be overcome with AXL or mTOR inhibitors. Likewise, Li et al. [165] reported that the complementary activated mTOR pathway facilitates resistance to WEE1 inhibitors. Thus, the dual inhibition of WEE1 and mTOR will induce extensive DNA replication stress, resulting in replication fork stalling, DNA damage, and cell death. Furthermore, Lewis et al. [166] found that cells with acquired resistance to adavosertib following its treatment have high MYT1 expression levels compared to sensitive cells. The suppression of MYT1 promoted CDK1 activity and helped overcome adavosertib resistance.

### Resistance to CHK1 inhibitors

Recently, it has been reported that the acquired resistance of prexasertib is associated with innate immunity [167, 168]. Blosser et al. [168] identified the correlation between prexasertib resistance with innate immunity genes and described the association between the sensitivity of prexasertib and the expression of E2F target genes. The expression of immune-related and E2F/G2M/SAC genes contributes to prexasertib resistance. The increased E2F/G2M/SAC expression and reduced replication stress or DNA damage are highly associated with NCI-H520 lung cancer cell resistance to prexasertib. Similarly, Manic et al. [169] confirm that an experimental increase of replication stress in advanced colorectal cancer cells eliminates the resistance to prexasertib. The decrease in oncogene-induced replication stress will reduce the stress generated by CHK1 inhibitors and lead to acquired drug resistance.

## The future of synthetic lethality

The recent discovery of PARP inhibitors leads to researches regarding genetic associations between potential therapeutic targets and cancer genes for BRCA1/2-mutated cancer patients [5]. However, the limits of sequence-based cancer genetic target discovery have almost been reached. Nonetheless, various targets for cancer drugs could still be discovered with technological advances. CRISPR-based genomic screenings can be used in various ways to deliver the next generation of targeted therapies, and address the loss of non-autonomous cell pathways, tumor suppressor genes, and unmarked oncogenes [170]. Since solid tumors are generally powered by a variety of driver mutations, it is possible to design combination therapies that can simultaneously address multiple distinct driver effects. The concurrent evaluation of multiple synthetic lethal interactions to discover new combination therapies still remains an underexplored aspect of synthetic lethality principle. Therefore, new technologies that are druggable and can expand the number of targets could potentially have a major impact on drug discovery.

The identification of synthetic lethal effects that are related to various tumor-specific genotypes has been brought to attention by synthetic lethality genotype-specific cell inhibition. For instance, KRAS oncogene mutations are common in various cancers and had been found that ATR or GATA2 transcription factor inhibition is synthetically lethal [171, 172]. Furthermore, mutations in the p53 gene are one of the most prevalent human tumor-specific genetic changes. Various potential synthetic lethal approaches that focus on p53-mutated tumor cells include the key determinant targeting of ATR replication fork stability [111], PI3-kinase signaling cascade components [121], WEE1 protein kinase [123], and CHK1/2 DNA damage checkpoint kinase [140]. Nonetheless, the validation and discovery approaches should emphasize the importance of the identification of synthetic lethal effects with substantial magnitude. Computational analysis improvements and the utilization of better screening technologies could result in novel synthetic lethal interactions which could be applied for better therapeutic targeting.

Recently, nanomedicine had become a promising tool for effective drug delivery, resulting in the reduction of drug dosage, adverse events, and off-target drug effects [173–175]. The emergence of nanomedicine based on synthetic lethality provides a new path for cancer treatment with enhanced efficacy, increased bioavailability, reduced toxicity, sustained drug release, and positive treatment outcomes [176–178]. This concept holds great potential for personalized nanotechnological-based chemotherapeutic treatment to achieve precise delivery of the regimen. Furthermore, the physicochemical properties of the nanoparticle could also be incorporated with DDR inhibitors to further enhance the treatment efficiency [177–179]. However, further preclinical and clinical studies are still required to be able to determine the most effective and clinically relevant treatment. The increase of cancer pathological understandings and targeting receptors, with the correlation of nanoparticle engineering, would undeniably enhance the efficiency of cancer therapy in the future.

## Conclusion

Synthetic lethality is a concept in genetics that have a significant impact on cancer study. The concept of synthetic lethality opens a new path for the development of cancer treatment by targeting the synthetic lethal targets after the cancer-specific mutations are identified. The identification of drug targets and genetic contexts, with increasingly powerful tools and applying synthetic lethality concepts, would definitely be an effective therapeutic option and a transformative opportunity for patients. The incessantly evolving CRISPR screening technology has the potential to address the heterogeneity of tumors in primary drug resistance and genetic mutations in secondary drug resistance. Multiple DDR inhibitors are being developed and tested in various stages, thus meticulous considerations of the mechanisms are needed to maximize the potential of these drugs. The lessons learned from PARP inhibitors have demonstrated that the challenges in managing toxicities resulted from combination therapies, yet clinicians should focus on both monotherapy and combination therapy to establish the finest therapeutic option for cancer patients. Despite the great potential of these methods, several obstacles remain in moving from discovering drug targets to utilizing these effective medicines clinically. Nevertheless, synthetic lethality paved a pathway to a wider range of possibilities with present and future applications.

## Data Availability

Data sharing not applicable to this article as no datasets were generated or analyzed during the current study.

## Abbreviations

PARP: Poly(ADP-ribose) polymerase
DDR: DNA damage response
ROS: Reactive oxygen species
NRF2: Nuclear factor erythroid 2–related factor 2
HSP90: Heat shock protein 90
CDK1/2: Cyclin-dependent kinase 1/2
PLK1: Polo-like kinase 1
RNAi: RNA interference
CRISPR: Clustered regularly interspaced short palindromic repeats
shRNA: Short hairpin RNA
siRNA: Small interfering RNA
SSB: Single-strand break
DSB: Double-strand break
PFS: Progression-free survival
HR: Homologous recombination
NHEJ: Non-homologous end joining
ATR: Ataxia telangiectasia and rad3 related
DNA-PK: DNA-dependent protein kinase
CHK1/2: Checkpoint kinase 1/2
BER: Base excision repair
APE1: Apurinic/apyrimidinic endonuclease 1
POLB: DNA polymerase b
XRCC1: X-ray repair cross-complementing
PD-L1: Programmed death-ligand 1
STING: Stimulator of interferon genes
CXCL10: C-X-C motif chemokine ligand 10
IFN-β: Interferon-beta
PD-1: Programmed cell death protein 1
CCL5: Chemokine ligand 5
cGAS: Cyclic GMP–AMP synthase
IFN-γ: Interferon-gamma
RPA: Replication protein A
ABCG2: ATP-binding cassette G2
pS6: Phosphorylated ribosomal S6
